# The Relationship of Amount of Resection and Time for Recovery of Bell’s Phenomenon after Levator Resection in Congenital Ptosis

**DOI:** 10.2174/1874364101711010024

**Published:** 2017-02-28

**Authors:** Ruchi Goel, Divya Kishore, Smriti Nagpal, Sparshi Jain, Tushar Agarwal

**Affiliations:** Department of Ophthalmology, Gurunanak Eye Center, New Delhi, India

**Keywords:** Ptosis, Bell`s, Levator resection, Keratitis, Lagophthalmos, Tarsorrhaphy

## Abstract

**Background:**

Recovery of Bell`s phenomenon after levator resection is unpredicatable. Delayed recovery can result in vision threatening corneal complications.

**Aim:**

To study the variability of Bell’s phenomenon and time taken for its recovery following levator resection for blepharoptosis and to correlate it with the amount of resection.

**Methods:**

A prospective observational study was conducted on 32 eyes of 32 patients diagnosed as unilateral simple congenital blepharoptosis who underwent levator resection at a tertiary care center between July 2013 and May 2015. Patients were followed up for 5 months and correction of ptosis, type of Bell`s, duration of Bell`s recovery and complications were noted.

**Results:**

The study group ranged from 16-25 years with 15:17 male: female ratio. There were 9 mild, 16 moderate and 7 severe ptosis. Satisfactory correction was achieved in all cases. Good Bell`s recovery occurred in 13 eyes on first post-op day, in 2-14 days in 19 eyes and 28 days in 1 eye. Inverse Bell`s was noted along with lid oedema and ecchymosis in 2 patients. Large resections (23-26mm) were associated with poor Bell`s on the first postoperative day (p=0.027, Fisher`s exact test). However, the duration required for recovery of Bell`s phenomenon did not show any significant difference with the amount of resection. (p=0.248, Mann Whitney test). Larger resections resulted in greater lagophthalmos (correlation=0.830, p<0.0001). Patients with recovery of Bell`s delayed for more than 7 days were associated with greater number of complications (p=0.001 Fisher`s Exact Test).

**Conclusion:**

Close monitoring for Bell`s recovery is required following levator resection.

## INTRODUCTION

Bell`s phenomenon is deviation of the eye, which accompanies an attempted closure of the eyelids [1]. It is a protective mechanism in which the eyes roll upward and outward on attempted bilateral voluntary eyelid closure against resistance. Great variability has been noted in Bell`s phenomenon in normal population. A person may have differences in direction and /or amplitude of eye movement between the two eyes or on different visits [2]. Inverse Bell’s is a variation in which the eye moves downwards and inwards on forceful closure [3].

Levator muscle resection is a standard procedure for ptosis correction and a good Bell`s phenomenon is a pre- requisite for ptosis surgery [4, 5]. Post- operative rehabilitation depends on the recovery of Bell`s phenomenon and varies from patient to patient.

To the best of our knowledge, except for a few case reports, none of the previous studies have correlated the amount of resection with the time required for recovery of Bell`s phenomenon. In this study, we shall study the effect of levator resection on recovery of Bell’s phenomenon and its association with post-operative complications.

## MATERIAL AND METHODS

A prospective observational study was conducted on 32 eyes of 32 patients of simple congenital blepharoptosis attending the Oculoplasty clinic of Guru Nanak Eye Centre, New Delhi, who underwent levator resection between July 2013 and May 2015. Inclusion criteria included age >5 years, unilateral ptosis, levator action ≥4mm and good Bell’s phenomenon. Patients with dry eye, diminished corneal sensations, lid abnormalities, previous ptosis surgery, strabismus or uncontrolled illness were excluded from the study.

Bell`s phenomenon was checked on attempted light closure of the lid after elevating the patient’s upper eyelids using the finger and thumb of one hand. Pre- operative work-up included general physical examination, best corrected visual acuity and cycloplegic refraction, marginal reflex distance, vertical palpebral aperture in upgaze, downgaze and primary position, horizontal palpebral aperture, ocular motility, corneal sensations and Schirmer Test for dry eye. A written informed consent, in accordance with the guidelines of the Declaration of Helsinki approved by the institute’s ethical committee was taken. Complete hemogram, bleeding and clotting time and random blood sugar was done.

All patients underwent LPS resection from the skin approach, under general or local anaesthesia depending on the patient`s age and were operated by a single surgeon (RG) with the amount of resection ranging from 18mm to 28mm. An inverse frost suture was passed in all patients using 5-0 silk at the end of the procedure. Satisfactory correction was taken to be ptosis correction within 1mm of the fellow eye at the end of 5^th^ post op week.

Topical lubricant in the form of Polyvinyl alcohol 1.4% four hourly was prescribed in the operated eye till the recovery of Bell`s phenomenon after which it was reduced to three times daily till one month. Systemically, adults received Tab.Ciprofloxacin 500mg twice daily (children received Cap Amoxycillin 40 mg/kg/day in divided doses) for 5 days, and Tab Serratiopeptidase 10mg thrice daily (5mg for children) for five days. Povidone iodine was applied over sutures twice daily till suture removal. Frost suture was put up at night and while sleeping during the day and was removed after recovery of Bell`s phenomenon.

Patients were followed up on day 1, 1 week and then every two weeks for a total of 5 months.

Postoperatively type of Bell`s, complications such as lid oedema, dry eye, ecchymosis, exposure keratopathy and lagophthalmos if any, were noted. Lagophthalmos was assessed by asking the patient to extend his neck and gently close his eyes. The amount of lagophthalmos was measured as the distance between the upper and lower lids. Postoperative Bells’ phenomenon was recorded as normal or inverse. It was graded as good, fair and poor depending upon the amount of cornea visible, less than one third, one third to half or more than half respectively. For statistical evaluation, the fair and poor Bell`s was clubbed together. SPSS software 20 was used for data evaluation.

## RESULTS

There were 15 males and 17 female patients in an age group ranging from 6 to 25 years with a mean age of 16.26 years. The right eye was affected in 12 patients and the left eye in 20 patients. Nine patients had mild ptosis, 16 had moderate and 7 patients had severe ptosis (Table **1**).

**Fig. (1):**
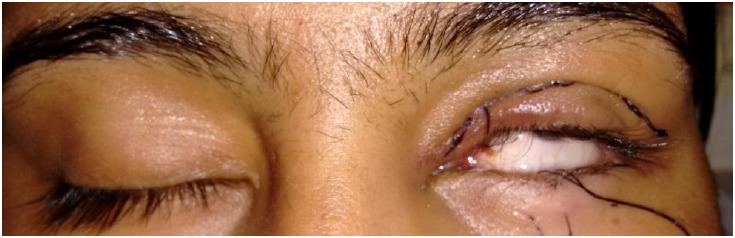
Clinical photograph of a patient showing a good Bell`s on the 1st post- operative day.

**Fig. (2):**
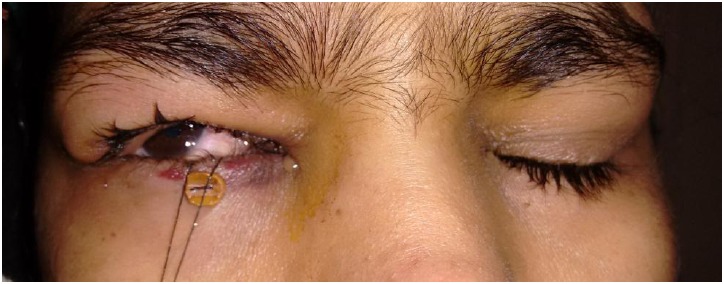
Clinical photograph of a patient showing a poor Bell's on the 1^st^ post- operative day.

**Fig. (3):**
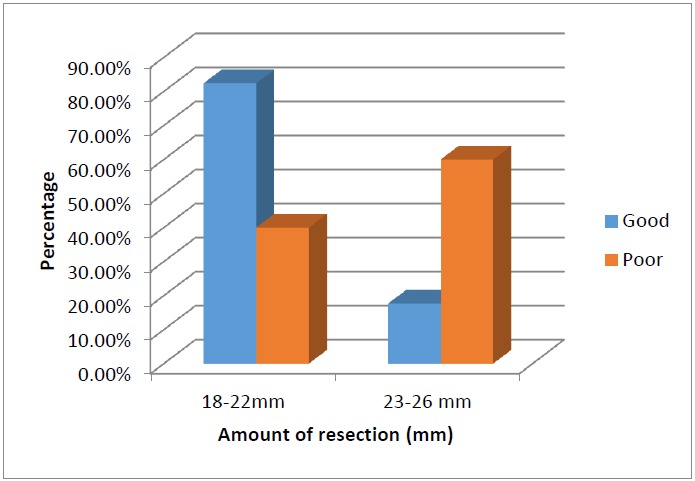
Bar chart showing the relationship between the amount of levator resection and Bell`s phenomenon on 1st post-operative day.

All the patients achieved satisfactory correction. In 13 patients Bells’ phenomenon was good on the first post-op day (Fig. **1**). Of the remaining, in 19 patients it took 2-14 days for good Bells’ to recover. (Fig. **2**) In majority of these patients, Bells recovered within the first week. In two of the patients inverse Bells was noted along with lid oedema and ecchymosis. One patient had poor Bells for 14 days and developed exposure keratopathy despite the presence of frost suture. He subsequently underwent paramedian tarsorrhaphy and the Bells’ phenomenon finally recovered on day 28. The patient had residual corneal opacity after healing of the keratitis. Large resections 23-26mm were associated with poor Bell`s on the first postoperative day. (p=0.027, Fisher`s exact test) (Fig. **3**).

**Fig. (4):**
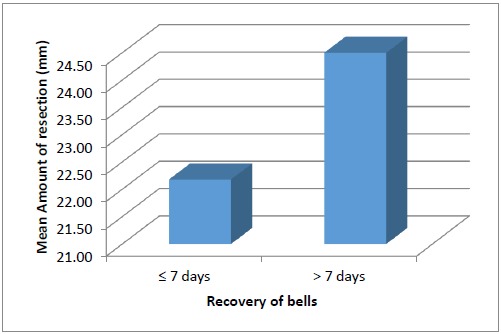
Bar chart showing relationship between the amount of levator resection and duration of Bell`s recovery in days.

The duration required for recovery of Bell`s phenomenon did not show any statistically significant difference with the amount of resection. (p=0.248, Mann Whitney test) (Fig. **4**).

**Fig. (5):**
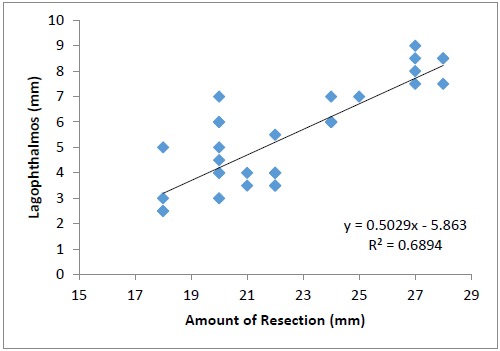
Scatter plot showing a strong relationship between the amount of levator resection and the amount of lagophthalmos.

**Fig. (6):**
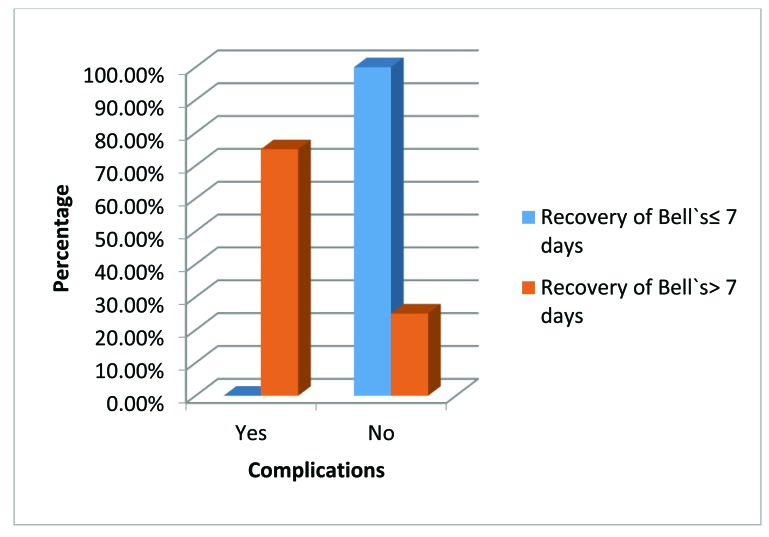
Bar chart showing relationship between the occurrence of complications and duration of Bell`s recovery.

Larger resections resulted in greater lagophthalmos (correlation=0.830, p<0.0001) (Fig. **5**). Patients with recovery of Bell`s delayed for more than 7 days were associated with greater number of complications like ecchymosis, lid edema and exposure keratopathy. (p=0.001, Fisher`s Exact Test) (Fig. **6**).

## DISCUSSION

Levator resection acts as a surgical trauma and leads to delayed recovery of Bell`s phenomenon on eye lid closure, predisposing the patient to corneal complications. The Bell`s phenomenon is an oculogyric phenomenon first described by Sir Charles Bell in 1823 in a case of lower motor neuron facial palsy [6]. It is a protective response that stabilizes the tear film and protects the ocular surface [7] and is absent in 10% of the normal population [8]. The exact mechanism of this response is unknown. Brainstem pathways between the 7^th^ nerve nucleus in pons and the third cranial nerve nuclear complex in the rostral midbrain are suggested. In patients with inability to voluntarily elevate their eyes, presence of Bell’s phenomenon further strengthens this hypothesis. According to Hiraoka, the mesncephlic reticular nucleus links the pathways controlling bilateral lid closure and upward movement of both the eyes [9]. Under the influence of the cortical and subcortical regions, when the orbicularis oculi (supplied by 7^th^ nerve) contracts, the superior rectus and inferior oblique muscles (supplied by 3^rd^ nerve) also contract and act synergistically to move the eye upward, causing normal Bell’s phenomenon [10].

In our study, the Bell`s recovered by 1 week in most of the patients with the longest recovery time being 4 weeks. Betharia et al found that even on moderate resection gross reduction in the Bell's phenomenon can occur in immediate postoperative period despite good cosmetic appearance and minimal lagophthalmos [11]. We also had similar observations. However, the larger resections were associated with poor Bell`s and greater lagophthalmos in our series. Though delayed recovery has been attributed to residual ptosis, in ptosis with myopathy and congenital complicated ptosis [11], it occurred in 4 eyes in our study in absence of these factors. Also, we did not observe any statistically significant correlation between the amount of resection and duration of recovery. (p=0.248, Mann Whitney test).

In Inverse Bell’s phenomenon, the eye moves downwards on lid closure. It has also been proposed that there are connections between 4^th^ and 7^th^ nerve nuclei though not as well established and numerous as those between 3^rd^ and 7^th^ nerve nuclei. But due to some pathologic conditions, connections between 4^th^ and 7^th^ nerve come into play and the mechanism of Bell’s phenomenon is reversed resulting in eye moving downwards due to action of superior oblique and inferior rectus. It probably occurs as a protective phenomenon where the up response fails to provide protection to the ocular surface [10]. Inverse Bell’s phenomenon was seen in 2 of our patients. In both the cases, large resection had been performed and the patients had postoperative lid edema and ecchymosis. This was in accordance with the earlier published reports [3, 12], where severe edema and hyperemia of the superior fornix had been attributed to aggravate the relationship between eyelid movements, superior rectus movements and normal Bell`s phenomenon. The period of resolution coincided with the recovery of Bell`s.

Lagophthalmos, is another concern especially in the presence of poor Bell`s. The combination of lagophthalmos and poor Bell`s has been known to make management of myopathic ptosis challenging [13]. However, similar problems can also occur in ptosis correction of more than 5.5 mm or lagophthalmos of more than 5.0 [7]. We also observed a strong correlation between the amount of resection and lagophthalmos. Exposure keratopathy occurred in one of our patients who had undergone a 28 mm resection and developed poor Bell`s which eventually recovered on 28^th^ postoperative day. This probably occurred due to improper taping of frost suture. The patient was managed by temporary paramedian tarsorraphy to facilitate recovery of Bell`s and healing of keratitis by preventing movement-induced micro trauma to the superior fornix by immobilization and stabilizing the tear film. Other available modalities of treatment for this complication are amniotic membrane grafts, soft bandage contact lenses and scleral contact lens [14]. The occurrence of keratitis is reported with inverse Bell`s and it has been postulated that lower eyelid being inadequate in providing protection as compared to the upper lid may result in exposure [15]. We did not observe any corneal complications in our patients with inverse Bell`s and this could be because of patient compliance in adequately taping the frost suture and instilling postoperative lubricating drops till recovery of normal Bell`s.

To conclude, in patients undergoing levator resection, a close watch is required on Bell`s recovery. Resections more than 23mm are especially prone to poor postoperative Bell`s and lagophthalmos, a combination that predisposes to exposure keratitis. Also, patients with delayed recovery of normal Bell`s warrant copious use of lubricants and if need be a temporary tarsorrhaphy to expedite recovery.

## Figures and Tables

**Table 1:** Preoperative and post-operative raw data showing the amount of ptosis and postoperative Bell's phenomenon.

**S.No**	**Amount of ptosis**	**VPA**	**MRD1**	**LPS action**	**Amount of resection(in mm)**	**Type of Bells’ on day 1**	**Recovery of bells’**	**Complications**	**Lagophthalmos (in mm)**
1	2	7	2	7	21	Good	1	-	4
2	3	6	1	6	20	Good	8		6
3	2	7	2	6	18	Good	1	-	5
4	3	6	1	5	24	Good	1	-	6
5	3	6	1	5	24	Inverse	11	Ecchymosis	6
6	3	6	1	6	22	Good	1	-	3-4
7	3	6	1	6	22	Good	1	-	3-4
8	3	6	1	6	22	Good	1	-	4
9	3	6	1	6	20	good	7		6
10	3	6	1	6	20	Good	2	-	4
11	2	7	2	7	20	Good	1	-	4-5
12	3	6	1	6	22	Good	1	-	4
13	3	6	1	6	22	Good	1	-	4
14	3	6	1	6	20	Good	1	-	4
15	4	5	0	4	27	Inverse	8	Lid oedema	7-8
16	2	7	2	7	21	Good	1	-	3-4
17	3	6	1	6	22	Good	1	-	5-6
18	3	6	1	6	20	Poor	5		7
19	4	5	0	4	27	Poor	28	Exposure keratopathy	8-9
20	3	6	1	5	24	Fair	3		7
21	2	7	2	8	18	Fair	2	-	2-3
22	2	7	2	7	20	Fair	3	-	3
23	4	5	0	4	28	Poor	7		7-8
24	2	7	2	7	20	Fair	3	-	5
25	4	5	0	4	27	Poor	6		8
26	2	7	2	8	18	Fair	2	-	2-3
27	4	5	0	4	28	Poor	6		8-9
28	3	6	1	5	25	Fair	3		7
29	4	5	0	4	28	Poor	7		8-9
30	4	5	0	4	27	Poor	7		9
31	3	6	1	5	24	Fair	3		6
32	2	7	2	8	18	Fair	2	-	3
